# ﻿*Epitomaptaaumakua* sp. nov., a new species of apodous sea cucumber from Hawai`i (Echinodermata, Holothuroidea, Apodida)

**DOI:** 10.3897/zookeys.1183.111620

**Published:** 2023-10-31

**Authors:** Francisco Alonso Solís-Marín, Carlos Andrés Conejeros-Vargas, Andrea Alejandra Caballero-Ochoa, Sheila Colleen Byers

**Affiliations:** 1 Colección Nacional de Equinodermos “Dra. Ma. Elena Caso Muñoz”, Laboratorio de Sistemática y Ecología de Equinodermos, Instituto de Ciencias del Mar y Limnología (ICML), Universidad Nacional Autónoma de México (UNAM), Mexico City, C.P. 04510, Mexico; 2 Posgrado en Ciencias del Mar y Limnología, Universidad Nacional Autónoma de México, Av. Ciudad Universitaria 3000, C.P. 04510, Coyoacán, Mexico City, Mexico; 3 Facultad de Ciencias, Universidad Nacional Autónoma de México. Circuito exterior s/n, Mexico City, C.P. 04510, Mexico; 4 Posgrado en Ciencias Biológicas, Instituto de Geología, Universidad Nacional Autónoma de México, C.P. 04510, Mexico City, Mexico; 5 Beaty Biodiversity Museum, University of British Columbia, Vancouver, Canada

**Keywords:** Leptosynaptinae, Synaptidae, taxonomy

## Abstract

*Epitomaptaaumakua***sp. nov.** occurs at a depth of 2.5 m in Kualoa, O`ahu, Hawai`i, living in coarse sand. It is distinctive in having 12 pinnate tentacles, each tentacle with three pairs of digits and 6–8 sensory cups. The body wall bears papillae or oval bumps, and the length of body reaches a maximum length of 18.4 mm after relaxation.

## ﻿Introduction

The apodous sea cucumbers of the subfamily Leptosynaptinae Smirnov, 1989 (Apodida Brandt, 1835; Synaptidae Burmeister, 1837) are interstitial organisms that inhabit intertidal and shallow waters. They have a vermiform, translucent integument (Hendler et al. 1995; Woo et al. 2021). Heding (1928) placed the genus *Epitomapta* Heding, 1928 in the subfamily Synaptinae and included *Epitomaptaroseola* (Verrill, 1873) from Connecticut and Massachussets, USA, and *E.tabogae* Heding, 1928 from Taboga and Taboguilla, Panama. Heding based the new genus on the presence of notched rather than perforated radial pieces of the calcareous ring. Later, Solís-Marín et al. (2019) described a new species from the Tropical East Pacific in Mexico, *E.simentalae* Solís-Marín, Conejeros-Vargas, Caballero-Ochoa & Arriaga-Ochoa, 2019, based on having 12 tentacles, with each tentacle with two or three pairs of digits and 4–6 sensory cups, and a body lacking papillae or oval bumps. *Epitomapta* is, thus, currently represented by four nominal species, including the new one described here. Smirnov (1989) placed the genus in the subfamily Leptosynaptinae.

## ﻿Materials and methods

Specimens are preserved in the Marine Invertebrate Collection of the Beaty Biodiversity Museum, University of British Columbia, Canada (MI). Ossicles were extracted from the body wall (anterior, medium, and posterior region), longitudinal muscles and one whole tentacle. The tissue was dissolved in household bleach (5.0–6.5%). Bleach was washed off from the ossicles by rinsing them twice with distilled water. Hereafter the distilled water was replaced by rinsing the ossicles with 70, 80, and 95% ethanol. Finally, absolute ethanol was added to the ossicles, whereafter a small aliquot was taken and placed to dry on a scanning electron microscope (SEM) stub. The dry sample was sputter coated with 5 nm gold/palladium (80/20) using a Leica EM ACE600 and imaged with a Zeiss Crossbeam 350 SEM.

## ﻿Taxonomy

**Order Apodida Brandt, 1835**

**Family Synaptidae Burmeister, 1837**

### ﻿Subfamily Leptosynaptinae

Taxon classificationAnimaliaApodidaSynaptidae

Smirnov, 1989

6CA65860-CDF0-5198-8F7E-BB08E990456C

#### Diagnosis.

Pinnate tentacles 10, 11 or 12, with 1–9 digits on each side. Digits increase in size from base to tip of tentacle. Anchor plate develops from a rod which lies at a right angle to stock of developing anchor. Anchor plates with small number of holes, usually seven (6+1) in main part of the plate: six holes form a circle around a central hole. Articular end of plate usually has a “ledge” for contact with anchor keel. Anchor arms regularly serrated, rarely smooth, and without minute knobs on the vertex (Heding 1928; Smirnov 1989).

### ﻿Genus Epitomapta

Taxon classificationAnimaliaApodidaSynaptidae

Heding, 1928

14A800E8-2D38-5997-A4AE-78AC4114F940

#### Diagnosis.

Tentacles pinnate, usually 12. Digits in 2–5 pairs on each side (rarely two or none). Sense organs never in form of pigment-eyes, but as minute cups on inner face of stalk of tentacles. Calcareous ring well developed. Radial pieces not perforated for passage of nerves, but with an anterior notch. Cartilaginous ring absent. Polian vesicle usually single. Stone canal single, unbranched. Ciliated funnels of different shapes and attached to body wall, not to mesenteries. Calcareous deposits in body wall are anchors, anchor plates and miliary granules; tentacles with rods only. Stock of anchors finely toothed, but not branched; arms usually with teeth on outer edge; vertex smooth. Anchor plates oval, with large central hole, surrounded by six large holes, usually more or less dentate, and two large and several small smooth holes at narrow posterior end, but without an arched bow crossing outer surface; broad end often with additional dentate holes (Solís-Marín et al. 2019).

#### Type species.

*Epitomaptatabogae* Heding, 1928 by original designation.

### ﻿ Epitomapta aumakua sp. nov.

Taxon classificationAnimaliaApodidaSynaptidae

1C2C3B9B-0210-5F7F-931C-FE41FB317306

https://zoobank.org/01CD60ED-A303-4C39-A4B7-CD0616E5FF08

Figs 1 , 2 , 3

#### Type materials.

***Holotype*.**MI 4942, 18.4 mm total length (TL), off Kualoa, O`ahu, Hawai`i, Pacific Ocean 21°30'N, 157°50'W, 2.5 m depth, July 1975. ***Paratypes*.**MI 4944, 2 specimens, 1 extensively dissected, same data as the holotype.

#### Type locality.

Off Kualoa, O`ahu, Hawai`i, Pacific Ocean 21°30'N, 157°50'W.

#### Diagnosis.

Body wall smooth, covered with small, oval-circular bumps, especially on anterior part of body. Tentacles 12, each with three pairs of lateral digits and a terminal digit; 6–8 sensory cups on each tentacle. Polian vesicle, 1/10 of body length; stone canal single, unbranched. Anchor and anchor plates of one kind: anchors usually exceeding 170 µm in length, plates exceeding 110 µm in length. Miliary granules scarce, only present in longitudinal muscles, relatively coarse, usually in form of simple flat, often faintly undulating, stout, straight rods with enlarged ends, slightly bent but never C-shaped, usually exceeding 20 µm in length. Tentacle ossicles shaped like smooth, flattened rods, not exceeding 50 µm in length, curved, with perforated ends; some rods broad (ca 14 µm in width) with few circular peripheral holes.

#### Holotype description.

**Figure 1.**
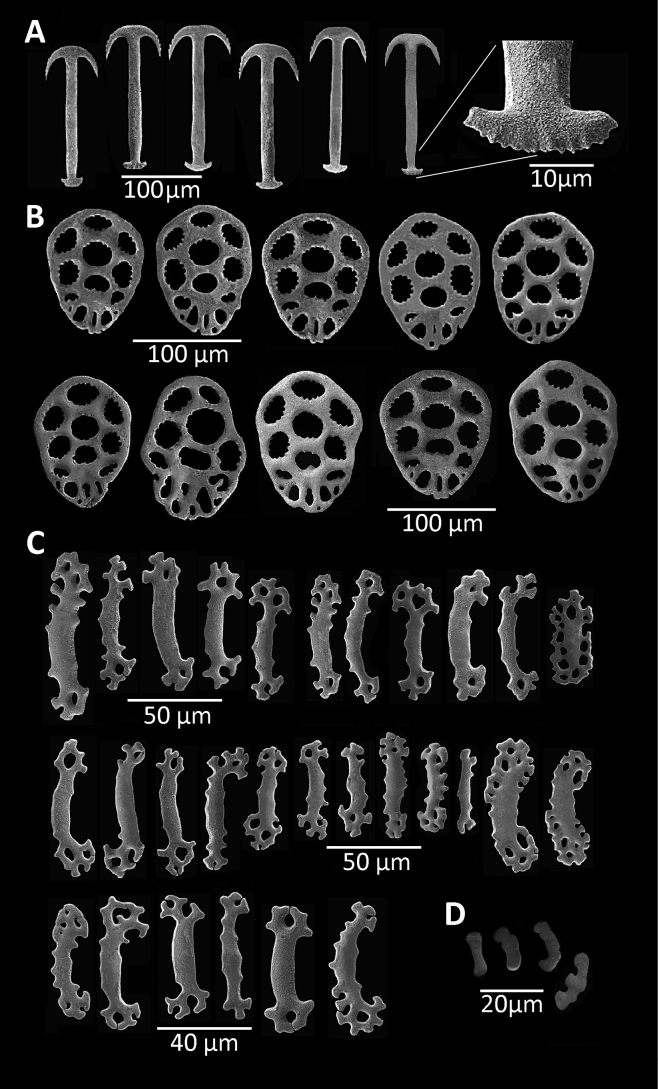
*Epitomaptaaumakua* sp. nov. Holotype MI 4942 **A** anchors from mid-body, showing the detail of the posterior part **B** anchor plates from mid-body **C** rods from tentacles **D** miliary granules from the body wall.

**Figure 2.**
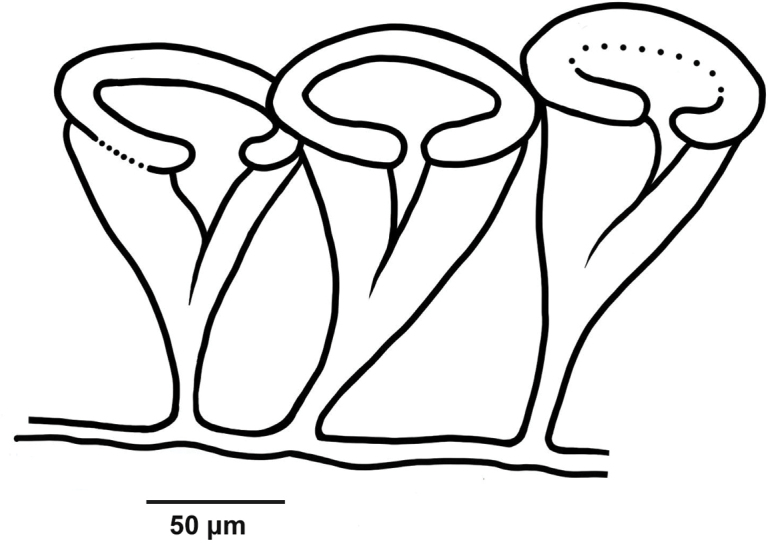
*Epitomaptaaumakua* sp. nov. Paratype MI 4944. Ciliated funnels showing their differing sizes and shapes.

**Figure 3.**
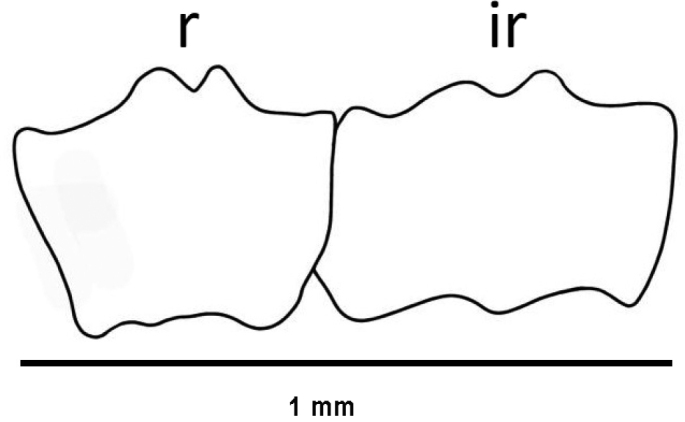
*Epitomaptaaumakua* sp. nov. Holotype MI 4942, calcareous ring. Abbreviations: r = radial piece, ir = interadial piece.

18.4 mm long. Specimen uniformly whitish, body wall translucent when expanded. Anchors (Fig. 1A) not projecting through body wall. Tentacles 12, each with three pairs of digits and a terminal digit; digits increase in length distally, and terminal digit longest. Inner (oral) surfaces of tentacles with double row of well-developed sensory cups; up to eight sensory cups on each tentacle. Ciliated funnels of various shapes (Fig. 2) occur on body wall, not on mesenteries. Two longitudinal rows of round-lipped, ciliated funnels present, each row attached to one side of one longitudinal muscle; a single V-shaped notch splits round lips of funnels and extends about 1/2 length of funnel. Polian vesicle single. Stone canal single, unbranched. Calcareous ring simple, stout, well developed (Fig. 3); radial pieces notched anteriorly, more conspicuous than that in interradial pieces; not pierced.

***Ossicles*.** Body-wall deposits comprise anchors and anchor plates (Fig. 1A, B). Anchors and plates at anterior, middle, and posterior body wall essentially similar. Anchors average 170 µm in length and 55 µm in largest width (width of the arms). Arms carry up to five conspicuous teeth; vertex smooth. Stock unbranched, but equipped with numerous small, sharp projections (Fig. 1A). Anchor plates elongate, approximately oval, with numerous toothed perforations. Anchor plates average 110 µm in length and 90 µm in greatest width (Fig. 1B). Miliary granules scarce, present only in epithelium covering longitudinal muscles, variable in shape but generally flat and tending to have enlarged endings. Granules up to ca 20 µm in length (Fig. 1D). Tentacle ossicles small (40–50 µm in length), smooth, shaped like flattened, curved rods, with perforated ends (Fig. 1C). Some rods flat and broad (ca 14–16 µm in width) having 6–10 circular peripheral holes.

***Paratype variations*.** Specimens range from 16–17 mm in length.

#### Etymology.

The specific epithet *aumakua* refers, in Hawaiian mythology, to a person or family god that originated as a deified ancestor, who takes on physical forms as spirit vehicles. Here it is used as a non-Latin noun in apposition.

#### Ecology.

*Epitomaptaaumakua* sp. nov. occurs at 2.5 m depth, buried in coarse sand.

#### Geographical distribution.

Known only from its type locality.

## ﻿Discussion

*Epitomaptaaumakua* sp. nov. is very similar to its central Eastern Pacific congener *E.simentalae* but differing in the number of sensory cups per tentacle (4–6 in *E.simentalae*, 6–8 in *E.aumakua* sp. nov.). In addition to the geographical distribution, *E.aumakua* sp. nov. is smaller (<20 mm) than *E.simentalae* (<50 mm) (Solís-Marín et al. 2019). *Epitomaptaaumakua* sp. nov. clearly differs from *E.tabogae* and *E.roseola* in the number of sensory cups per tentacle (8–14 in *E.tabogae*, 2–5 in *E.roseola*), and in the number of pairs of digits present on the tentacles (5–6 in *E.tabogae*, 7 in *E.roseola*, and 2–3 in *E.aumakua* sp. nov.). *Epitomaptatabogae* was originally recorded from Taboga and Taboguilla, Panama, by Heding (1928) and is distributed throughout the Gulf of California (Solís-Marín et al. 2009), whereas *E.aumakua* sp. nov. currently is known only from Hawai`i. In its original description (Verrill 1873), *E.roseola* was recorded from Long Island Sound, Connecticut, and Vineyard Sound, Massachusetts; it was subsequently described from Bermuda (Heding 1928) and later recorded from Connecticut and Massachusetts to Florida (USA) (Hendler et al. 1995). Most recently, *E.roseola* has been reported from the South American coast (Brazil) (Miranda et al. 2015). It has never been reported from Hawai`i.

The anchors of the body wall in *E.aumakua* sp. nov. are similar in shape to those of *E.roseola*, but they differ in size, being approximately 160–170 μm long and 76–80 μm wide in *E.aumakua* sp. nov. (Fig. 1A) versus 120–150 μm long and 70–75 μm wide in *E.roseola* (Heding 1928). The anchors of the posterior region of the body wall in both these species are similar and can reach up to 150 μm long and 70 μm wide; anchors from the anterior end of the body wall in *E.roseola* measure almost 120 μm long and 70 μm wide (Heding 1928), while in *E.aumakua* sp. nov. they are 90–150 μm long and 70 μm wide. On the other hand, the anchors of the Pacific *E.tabogae* are 200 μm in length and 100 μm in width in the posterior region of the body, and 170 μm length and 100 μm width in the anterior region of the body (Heding 1928). Today *E.tabogae* and *E.aumakua* sp. nov. possess the largest known anchors of any species in this genus.

*Epitomaptaaumakua* sp. nov. is clearly distinguished from other species of the genus in having extremely large anchors, a character that has been used to differentiate species of the genus by various authors (see Heding 1928 and Hendler et al. 1995).

### ﻿Key to species of the genus *Epitomapta*

**Table:** 

1	Papillae or oval bumps present all over the body wall	**2**
–	Papillae or oval bumps absent. With 2–3 pairs of tentacle digits, each tentacle with 4–6 sensory cups. Miliary granules in the shape of small, C- and O-shaped bodies; no papillae or oval bumps present on the body wall	***E.simentalae***
2	Atlantic Ocean. With 7 pairs of tentacle digits, each tentacle with 2–5 sensory cups. Anchors of body wall exceed 120 μm in length (up to 150 μm). Miliary granules in the shape of small, oval rings and very few C-shaped bodies	***E.roseola***
–	Pacific Ocean	**3**
3	Central America (Panama). With 5–6 pairs of tentacle digits, each tentacle with 8–14 sensory cups. Anchors of body wall exceed 120 μm in length (up to 200 μm). Miliary granules in the shape of oval rings and very few C-shaped bodies	***E.tabogae***
–	Eastern Indo Pacific (Hawai`i). With 3 pairs of tentacle digits, each tentacle with 6–8 sensory cups. Anchors of body wall exceed 150 μm in length. Scarce miliary granules in the shape of stout, flat rods	***E.aumakua* sp. nov.**

## Supplementary Material

XML Treatment for Leptosynaptinae

XML Treatment for Epitomapta

XML Treatment for Epitomapta aumakua

## ﻿Additional information

### Author ORCIDs

Francisco Alonso Solís-Marín https://orcid.org/0000-0001-5729-3316

Carlos Andrés Conejeros-Vargas https://orcid.org/0000-0003-0148-0475

Andrea Alejandra Caballero-Ochoa https://orcid.org/0000-0001-5520-3823

Sheila Colleen Byers https://orcid.org/0009-0003-7470-5193

### Data availability

All of the data that support the findings of this study are available in the main text.
